# BGD-Net: An Object–Background Decoupling Network for Oriented Object Detection in Complex Remote Sensing Scenes

**DOI:** 10.3390/s26165193

**Published:** 2026-08-17

**Authors:** Jiaxin Xu, Hua Huo, Aokun Mei, Chen Zhang

**Affiliations:** College of Information Engineering and Artificial Intelligence, Henan University of Science and Technology, Luoyang 471000, China; xujiaxin0523@163.com (J.X.); 240410040061@stu.haust.edu.cn (A.M.); 250410040094@stu.haust.edu.cn (C.Z.)

**Keywords:** remote sensing images, oriented object detection, complex backgrounds, object–background decoupling, hard background mining, contrastive learning

## Abstract

Oriented object detection in high-resolution remote sensing images remains vulnerable to object–background confusion in complex scenes, where visually similar background structures can produce high-confidence false positives. To address this problem, we propose BGD-Net, an object–background decoupling framework that suppresses confusing background responses from the feature, sample, and optimization levels. First, a Background-Decoupled Feature Module (BDFM) separately models object responses and background activations and performs residual feature purification before proposal generation. Second, Hard Background Mining (HBM) identifies high-confidence, low-overlap background RoIs that are most likely to cause false-positive detections and assigns them greater training emphasis. Third, an Object–Background Contrastive Suppression Loss (OBCS Loss) uses the mined hard backgrounds as targeted negative samples to enlarge the representation gap between true objects and confusing backgrounds in the RoI embedding space. Experiments on DOTA-v1.0 show that BGD-Net achieves 82.94% mAP@0.5, improving the Oriented R-CNN baseline by 2.07 percentage points while reducing Hard-FP by 38.48%. The method also achieves 98.47% mAP@0.5 on HRSC2016 and 68.35% mAP@0.5 on DIOR-R, demonstrating effective performance across different remote sensing scenes. Ablation, multi-seed, and threshold-sensitivity experiments further verify the complementary contributions and reproducibility of the proposed components. BGD-Net introduces only a modest increase in model complexity, indicating a favorable balance between detection accuracy and practical efficiency.

## 1. Introduction

In recent years, oriented object detection has made significant progress in high-resolution remote sensing image interpretation. Two-stage detectors usually achieve high localization accuracy by generating rotated proposals and aligning regional features. For example, RoI Transformer [1] learns a spatial transformation to convert horizontal RoIs into rotated RoIs, thereby improving feature alignment for arbitrarily oriented objects. Oriented R-CNN [2] designs a lightweight oriented RPN and combines it with Rotated RoIAlign to achieve efficient oriented object detection. In contrast, one-stage detectors pay more attention to detection efficiency. R3Det [3] alleviates the mismatch between rotated bounding boxes and features through a feature refinement mechanism, while S^2^A-Net [4] improves oriented object representation by using feature alignment and orientation-aware detection modules. In addition, to address angle periodicity, boundary discontinuity, and parameter coupling in rotated box regression, CSL [5], DCL [6], GWD [7], and KLD [8] improve regression stability from the perspectives of angle encoding, label smoothing, and Gaussian geometric measurement. Multi-scale feature fusion structures, such as FPN [9], PANet [10], NAS-FPN [11], BiFPN [12], and ASFF [13], have also been widely used to improve the detection of small and multi-scale objects in remote sensing images.

Although existing methods have achieved promising performance in orientation modeling, feature alignment, multi-scale fusion, and rotated box regression, the object–background confusion problem in complex scenes remains insufficiently addressed [14,15]. High-resolution remote sensing images often contain background structures whose visual patterns resemble those of real objects [16,17]. For example, dock edges and shorelines may be confused with ships, runway markings may interfere with aircraft detection, and road or industrial structures may trigger false-positive responses. Such object-like background patterns can lead to high-confidence false positives in complex scenes [18,19].

To address the above issues, this paper proposes BGD-Net, an object–background decoupled learning network for oriented object detection in complex remote sensing scenes. Different from existing methods that mainly improve detection performance through orientation modeling, multi-scale feature enhancement, or rotated box regression optimization, this work focuses on decoupling object features from background interference under complex background conditions. Specifically, we first construct a Background-Decoupled Feature Module (BDFM) on the multi-scale features output by FPN. BDFM separately models object-related regions and background interference regions through an object-response branch and a background-suppression branch, and enhances object responses while weakening background activations through a residual background purification mechanism. Second, considering that ordinary negative samples cannot sufficiently represent complex background interference, we design a Hard Background Mining (HBM) strategy to automatically select high-confidence and low-overlap confusing background samples according to foreground confidence and rotated IoU. Finally, we propose an Object–Background Contrastive Suppression Loss (OBCS Loss), which pulls same-class object features closer and pushes hard background features away in the feature space, thereby improving the discriminative ability of the model against complex background interference.

The main contributions of this paper are summarized as follows:

- We propose BGD-Net, an object–background decoupled learning framework for oriented object detection in complex remote sensing scenes. It starts from the object–background coupling problem and reduces high-confidence false positives caused by complex background regions.
- We design a Background-Decoupled Feature Module (BDFM), which separately models object regions and background interference regions through an object-response branch and a background-suppression branch, achieving object enhancement and background purification at the feature level.
- We propose a Hard Background Mining (HBM) strategy, which automatically selects high-confidence and low-overlap confusing background samples according to foreground confidence and rotated IoU, enabling the model to focus more on hard backgrounds that are likely to cause false positives.
- We introduce an Object–Background Contrastive Suppression Loss (OBCS Loss), which pulls same-class object features closer and pushes hard background features away in the feature space, improving the discriminative ability of the model from the optimization perspective.

## 2. Related Work

### 2.1. Oriented Object Detection in Remote Sensing Images

Oriented object detection introduces angle information to provide a tighter geometric description for arbitrarily oriented objects. It can reduce redundant background regions caused by horizontal bounding boxes and alleviate overlap among densely distributed objects. Therefore, it has been widely applied to high-resolution remote sensing image interpretation [20,21,22]. Existing methods mainly focus on three aspects: rotated bounding box representation, regional feature alignment, and angle regression optimization. Representative techniques include rotated proposal generation [23], rotated RoI feature extraction [1], feature refinement [3], rotation-equivariant modeling [24], angle encoding [5,6], and Gaussian distribution-based measurement [7,8]. These methods effectively improve localization accuracy and regression stability for arbitrarily oriented objects.

Recent oriented object detectors further improve feature representation, proposal generation, and localization. Gliding Vertex [25] represents oriented objects by regressing vertex offsets from horizontal boxes, while ReDet [24] introduces rotation-equivariant representations. DODet [26] and AOPG [27] improve feature alignment and oriented proposal generation, respectively. KFIoU [28] optimizes rotated-box regression through Gaussian-based overlap modeling, whereas LSKNet [29] enhances remote-sensing feature representation using large selective kernels. More recent methods, including Oriented RepPoints [30], DCFL [31], and RQFormer [32], explore point-based representation, dynamic sample learning, and end-to-end query modeling.

However, most existing studies mainly focus on the geometric modeling of oriented objects, including representation, feature alignment, and rotated-box regression [33,34,35]. Comparatively less attention has been paid to explicitly separating object responses from confusing background responses in complex scenes. Therefore, this work shifts the focus from rotated-object geometry alone to object–background decoupling, aiming to improve the discriminative capability of oriented detectors under complex backgrounds.

### 2.2. Feature Purification and Object–Background Disentanglement

Wenti1 Attention mechanisms improve feature representation by adaptively reweighting channels, spatial locations, or contextual responses. Representative methods such as SE-Net [36] and CBAM [37] enhance discriminative features through channel and spatial attention, while non-local and global-context modules capture long-range dependencies [38]. In remote sensing detection, such mechanisms have also been used to suppress irrelevant responses and strengthen object-related features [18,19,39].

More directly related to the object–background confusion considered in this work, feature purification and denoising have been investigated for cluttered aerial scenes. SCRDet jointly employs a supervised pixel attention network and a channel attention network to suppress noise and highlight object-related features, thereby improving the detection of small and cluttered rotated objects [40]. SCRDet++ further introduces instance-level feature denoising to reduce feature contamination caused by surrounding backgrounds and neighboring objects [41]. These studies demonstrate that explicitly reducing background contamination is important for robust oriented object detection in cluttered remote sensing scenes.

BGD-Net differs from these methods in the way background interference is modeled and exploited during optimization. BDFM explicitly maintains an object-response branch and a background-suppression branch, and the background branch is supervised by hard-background regions mined according to foreground confidence and rotated IoU. Moreover, these hard backgrounds are reused by OBCS Loss as targeted negatives in the RoI embedding space. Thus, the proposed framework links feature purification, proposal-level hard-background mining, and contrastive suppression in a unified training pipeline.

### 2.3. Hard Example Mining and Adaptive Sample Selection

In object detection, foreground and background samples are usually highly imbalanced. A large number of easy background samples can dominate the training process, while the samples that truly affect detection performance are often a small number of hard background samples. OHEM [42] selects high-loss samples online for training, enabling the model to focus more on hard-to-classify regions. Focal Loss [43] reduces the weights of easy samples and increases the contribution of hard samples, thus alleviating sample imbalance in dense detection. These methods improve the utilization of hard samples, but they usually select samples mainly according to classification loss or prediction confidence. They lack targeted modeling for high-confidence and low-overlap false-positive regions in complex remote sensing backgrounds.

In complex remote sensing scenes, some background regions are negative samples, but they are highly similar to real objects in texture, edge, or geometric structure. As a result, they can obtain high foreground confidence in the classification branch. Traditional detection losses mainly distinguish foreground and background at the classification-probability level, and cannot explicitly constrain the distance between object features and hard background features in the embedding space. Contrastive learning improves the discriminability of the feature space by pulling similar samples closer and pushing dissimilar samples apart. Representative methods, such as SimCLR, MoCo, and supervised contrastive learning, improve visual representation from the perspectives of data augmentation, dynamic negative-sample dictionaries, and category supervision, respectively [44,45,46]. Recently, contrastive learning has also been introduced into object detection to enhance instance-level feature representation and improve inter-class separability [47,48,49].

Based on the above analysis, this paper combines hard background mining with contrastive learning. First, high-confidence and low-overlap hard background samples are selected according to the foreground confidence and rotated IoU of candidate regions. Then, these samples are introduced into the Object–Background Contrastive Suppression Loss as key negative samples. Compared with randomly selected background negatives, hard background samples are closer to real false-positive sources. They encourage the model to learn fine-grained differences between real objects and confusing backgrounds, thereby improving object–background discriminability in complex remote sensing scenes.

### 2.4. Detector-Specific Contrastive Learning

Contrastive learning learns discriminative representations by encouraging semantically related samples to remain close while separating dissimilar samples in the embedding space. Representative image-level approaches include SimCLR, MoCo, and supervised contrastive learning [44,45,46]. However, directly applying image-level contrastive objectives to object detection does not fully account for detector-specific representations such as proposals and RoI features.

Recent studies have therefore incorporated contrastive learning into object detection. DetCo introduces hierarchical global-to-local contrastive objectives during self-supervised pre-training to improve representations for downstream detection tasks [48]. FSCE further applies contrastive proposal encoding to RoI features and encourages intra-class compactness and inter-class separability for few-shot object detection [47]. These methods demonstrate the importance of constructing contrastive objectives according to detector-specific representation levels.

The proposed OBCS Loss differs from existing detector-specific contrastive approaches in both sample construction and optimization objective. Rather than performing contrastive pre-training as in DetCo [48], OBCS is jointly optimized with the oriented detector under fully supervised training. Moreover, unlike FSCE, which focuses primarily on proposal-level category discrimination in few-shot detection [47], OBCS specifically uses high-confidence and low-overlap background RoIs mined by HBM as targeted negative samples. By directly enlarging the feature-space margin between real object RoIs and background regions most likely to produce high-confidence false positives, OBCS provides a task-specific contrastive suppression mechanism for object–background confusion in complex remote sensing scenes.

## 3. Method

### 3.1. Overall Framework

To alleviate the strong coupling between object responses and background interference in complex remote sensing scenes, this paper proposes a background-decoupled oriented object detection network, namely BGD-Net. Different from existing methods that mainly focus on rotated box representation, orientation modeling, or feature alignment, the core objective of BGD-Net is to reduce high-confidence false positives caused by complex backgrounds from the feature, sample, and optimization levels. Overall, the proposed method consists of three key components: the Background-Decoupled Feature Module (BDFM), the Hard Background Mining (HBM) strategy, and the Object–Background Contrastive Suppression Loss (OBCS Loss).

**Figure 1:**
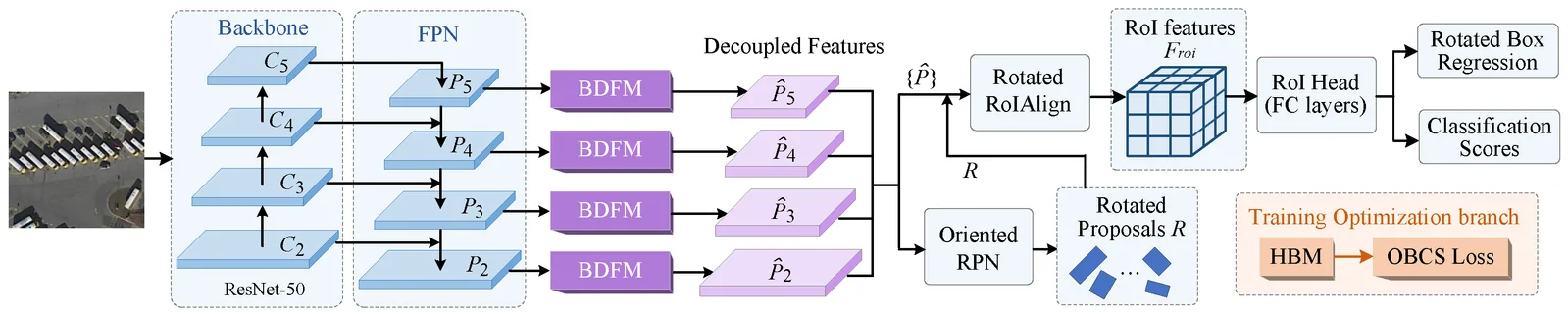
Overall framework of BGD-Net.

As shown in Figure 1, BGD-Net adopts a two-stage oriented object detection framework. Given an input image $I\in R^{3\times H\times W}$, the backbone network first extracts multi-level features $\left\{C_{2},C_{3},C_{4},C_{5}\right\}$. Then, the Feature Pyramid Network (FPN) fuses features from different levels and produces multi-scale feature representations $\left\{P_{2},P_{3},P_{4},P_{5}\right\}$. A conventional FPN mainly performs cross-scale semantic fusion, but does not explicitly distinguish object responses from background interference. Therefore, BDFM is introduced after FPN to enhance object-related features and purify background interference. The purified features $\left\{{\hat{P}}_{2},{\hat{P}}_{3},{\hat{P}}_{4},{\hat{P}}_{5}\right\}$ are fed into the oriented Region Proposal Network (oriented RPN) to generate rotated candidate regions R. Rotated Region of Interest Align (Rotated RoIAlign) is then used to extract RoI features $F_{roi}$, and the RoI Head performs classification and rotated box regression.

During training, HBM is further designed to select hard background samples with high foreground confidence and low rotated IoU from candidate regions. These samples are used as key negative samples in OBCS Loss. Therefore, BGD-Net not only suppresses background responses during forward propagation, but also explicitly enlarges the feature distance between real objects and hard backgrounds during optimization.

### 3.2. Background-Decoupled Feature Module

**Figure 2:**
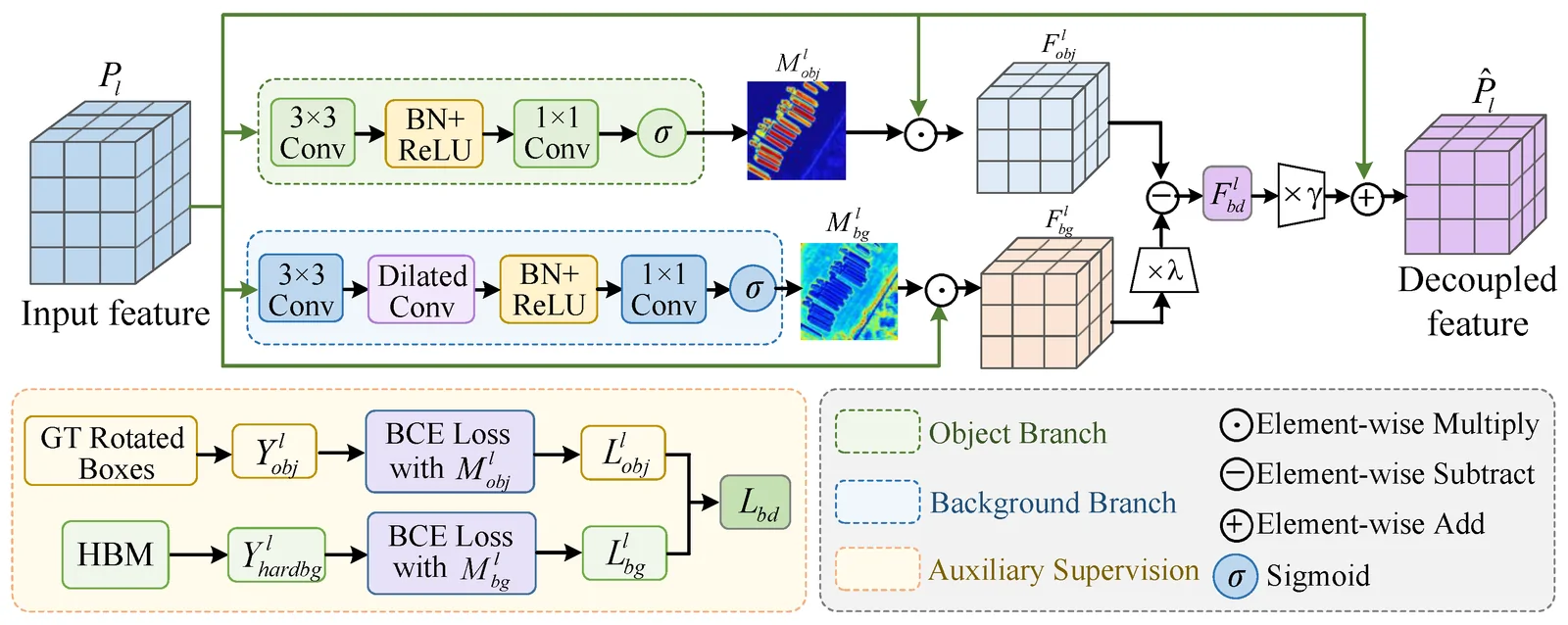
Structure of the Background-Decoupled Feature Module (BDFM).

Conventional FPN features jointly encode object responses and background activations, leaving the subsequent RPN and RoI Head to distinguish them mainly through classification supervision. To reduce this feature-level coupling before proposal generation, BDFM is inserted between the FPN and the oriented RPN to purify the multi-scale feature representations, as illustrated in Figure 2.

Unlike conventional attention mechanisms that generate a single weight map, BDFM constructs an object-response branch and a background-suppression branch simultaneously. These two branches learn object-related regions and background-interference regions separately, thereby achieving explicit object–background decoupling.

For the *l*-th FPN feature $P_{l}\in R^{C\times H_{l}\times W_{l}}$, BDFM contains two parallel branches: the object-response branch and the background-suppression branch. The object-response branch estimates the response intensity of each spatial location belonging to an object region:

(1) $$M_{obj}^{l}=\sigma \left(f_{obj}^{l}\left(P_{l}\right)\right),\;M_{obj}^{l}\in R^{1\times H_{l}\times W_{l}}.$$

where $M_{obj}^{l}$ denotes the object-response map, σ(·) denotes the Sigmoid function, and $f_{obj}^{l}\left(\cdot \right)$ is a lightweight convolutional mapping function.

The background-suppression branch estimates the response intensity of each spatial location belonging to a confusing background region:

(2) $$M_{bg}^{l}=\sigma \left(f_{bg}^{l}\left(P_{l}\right)\right),\;M_{bg}^{l}\in R^{1\times H_{l}\times W_{l}}.$$

where $M_{bg}^{l}$ denotes the background-suppression map, which is used to characterize the confounding background response that needs to be suppressed. $f_{bg}^{l}\left(\cdot \right)$ is the convolutional mapping function of the background branch. Both branches adopt a lightweight structure:

(3) $$\begin{array}{c} 3\times 3\;\mathrm{Conv}\to \mathrm{BN}\to \mathrm{ReLU}\to 1\times 1\;\mathrm{Conv}\to \mathrm{Sigmoid},\\ 3\times 3\;\mathrm{Conv}\to \mathrm{Dilated}\;\mathrm{Conv}\to \mathrm{BN}\to \mathrm{ReLU}\to 1\times 1\;\mathrm{Conv}\to \mathrm{Sigmoid}. \end{array}$$

This design obtains the spatial distributions of object responses and background responses with only a small number of additional parameters.

#### 3.2.1. Background-Decoupled Feature Generation

According to the object-response map and the background-suppression map, the object-enhanced feature and background-interference feature are obtained as follows:

(4) $$F_{obj}^{l}=P_{l}⊙M_{obj}^{l},\;F_{bg}^{l}=P_{l}⊙M_{bg}^{l}.$$

where ⊙ denotes element-wise multiplication, and the response maps are broadcast along the channel dimension. Then, the background-decoupled feature is generated through differential modeling:

(5) $$F_{bd}^{l}=F_{obj}^{l}-\lambda_{bg}F_{bg}^{l}.$$

where $\lambda_{bg}$ denotes the background suppression coefficient that controls the contribution of the background-response feature $F_{bg}^{l}$ during feature purification.

Considering that remote sensing images contain many small objects, directly suppressing background regions may accidentally weaken some weak object features. Therefore, a residual background purification mechanism is adopted:

(6) $${\hat{P}}_{l}=P_{l}+\gamma F_{bd}^{l}.$$

where γ is a learnable residual coefficient initialized to 0 and jointly optimized with the network parameters during training. It adaptively controls the contribution of the background-decoupled feature $F_{bd}^{l}$ to the original feature $P_{l}$.

Finally, BDFM outputs the background-purified multi-scale features $\left\{{\hat{P}}_{2},{\hat{P}}_{3},{\hat{P}}_{4},{\hat{P}}_{5}\right\}$. These features are then fed into Oriented RPN for proposal generation. Since background interference is weakened before candidate region generation, the false-positive pressure faced by the subsequent RoI Head is also reduced.

#### 3.2.2. Auxiliary Supervision

To make the learning of the object-response map and background-suppression map more stable, background-decoupled auxiliary supervision is introduced. First, an object-region mask $Y_{obj}^{l}$ is generated according to the ground-truth rotated boxes, where pixels inside rotated boxes are set to 1 and other locations are set to 0. The ordinary background mask can be written as

(7) $$Y_{bg}^{l}=1-Y_{obj}^{l}.$$

The object-response branch is supervised by the binary cross-entropy loss:

(8) $$L_{obj}^{l}=\mathrm{BCE}\left(M_{obj}^{l},Y_{obj}^{l}\right).$$

It should be noted that the ordinary background mask $Y_{bg}^{l}$ is not directly used to supervise the background-suppression branch. The reason is that ordinary background regions contain many easy background samples. If all background regions are supervised equally, the model may overemphasize ineffective background responses and fail to highlight the truly confusing backgrounds that cause false positives. During training, HBM selects hard background samples from the candidate regions in the current batch and projects their rotated boxes onto the corresponding FPN feature maps to generate the hard-background mask $Y_{\mathrm{hardbg}}^{l}$. Specifically, for an FPN level with stride $s_{l}$, the center coordinates and side lengths of each rotated box are scaled by $1/s_{l}$, while its orientation angle is preserved. The projected rotated box is then converted into its four corner points and rasterized as a binary polygon mask. Pixels covered by hard-background regions are assigned 1 and the remaining pixels are assigned 0; overlapping regions are merged by a logical OR operation. Based on this mask, the background supervision loss is defined as

(9) $$L_{bg}^{l}=\mathrm{BCE}\left(M_{bg}^{l},Y_{hardbg}^{l}\right).$$

The auxiliary supervision loss of BDFM is finally defined as

(10) $$L_{bd}=\sum_{l=2}^{5}\left(L_{obj}^{l}+L_{bg}^{l}\right),$$

Through this differentiated supervision, the object-response branch is guided to focus on real object regions, while the background-suppression branch is guided to focus on hard background regions that are likely to cause false positives. This prevents the two branches from learning similar or redundant response maps.

### 3.3. Hard Background Mining Strategy

**Figure 3:**
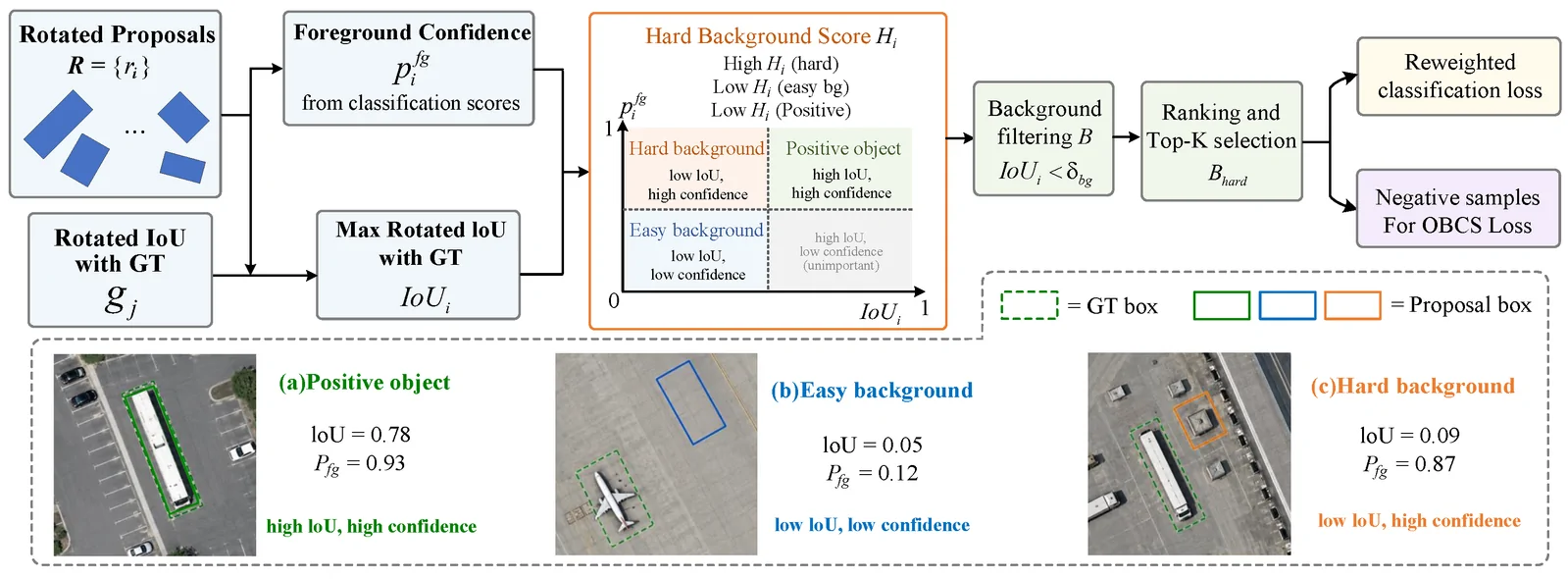
Hard background mining process of HBM. For each candidate region, HBM calculates the hard background score according to its foreground confidence and rotated IoU with the ground-truth boxes, and selects candidate regions with high confidence and low overlap as hard background samples.

In object detection training, most background RoIs are easily classified and provide limited discriminative information. HBM therefore focuses on a small subset of high-risk background regions that receive high foreground confidence while maintaining low rotated overlap with ground-truth objects. These regions are subsequently used for classification reweighting and contrastive optimization, as illustrated in Figure 3. Different from random negative sampling, HBM focuses on regions that “look like objects but are not objects”.

Let $R={\left\{r_{i}\right\}}_{i=1}^{N_{R}}$ denote the set of sampled rotated RoIs obtained after proposal generation and RoI sampling. For each $r_{i}$, the RoI classification branch predicts the class probabilities $p_{i}=\left(p_{i,0},p_{i,1},\ldots ,p_{i,C}\right)$, where $p_{i,0}$ denotes the background probability. Its foreground confidence is defined as $p_{i}^{fg}=\max_{c=1,\ldots ,C}p_{i,C}$, where *C* denotes the number of foreground categories.

The maximum rotated IoU between this candidate region and all ground-truth boxes is defined as

(11) $$IoU_{i}=\max_{j}IoU_{rot}\left(r_{i},g_{j}\right),$$

where $g_{j}$ denotes the *j*-th ground-truth rotated box, and $IoU_{rot}\left(\cdot \right)$ denotes rotated IoU. The hard background score is defined as

(12) $$H_{i}=p_{i}^{fg}\left(1-IoU_{i}\right).$$

When a candidate region has high foreground confidence but low overlap with real objects, $H_{i}$ becomes large, indicating that this region is more likely to be a hard background sample. This definition matches the typical characteristics of false positives in complex remote sensing scenes: they are easily regarded as objects by the model, but do not match any real object.

According to the rotated IoU threshold, background candidates are first selected as

(13) $$B=\{r_{i}|IoU_{i}<\delta_{bg}\}.$$

where $\delta_{bg}$ is the background threshold and is usually set to 0.3. Then, the background candidates are sorted according to the hard background score $H_{i}$, and the top-*K* samples are selected as the hard background set:

(14) $$B_{hard}=\mathrm{TopK}\left(B,H_{i}\right).$$

These hard background samples have two roles. First, they are used to reweight the classification loss, enabling the model to focus more on confusing backgrounds. Second, they are used as key negative samples in OBCS Loss for object–background contrastive learning. To enhance the learning of hard backgrounds, the classification loss is reweighted as

(15) $$L_{cls}^{hbm}=\sum_{i}\left(1+\alpha H_{i}\right)L_{cls}^{i}.$$

where $L_{cls}^{i}$ denotes the classification loss of the *i*-th RoI, and α is the hard-background weighting coefficient. For samples selected in $B_{hard}$, the classification weight is $\left(1+\alpha H_{i}\right)$, whereas $H_{i}$ is set to 0 for the remaining RoIs so that their classification weights remain unchanged. Therefore, hard-background samples with larger $H_{i}$ receive greater emphasis during training.

### 3.4. Object–Background Contrastive Suppression Loss

The classification loss distinguishes foreground and background at the label level, but it does not directly constrain their separation in the RoI embedding space. Consequently, hard background RoIs may remain close to object representations even after classification training, resulting in high-confidence false positives during inference. To address this issue, we propose the Object–Background Contrastive Suppression Loss (OBCS Loss), which uses the hard background samples mined by HBM as targeted negative samples. OBCS pulls same-class object features closer while pushing object features away from hard background features, thereby improving object–background discriminability in the embedding space.

For the *i*-th candidate region feature $F_{roi}^{i}$ output by the RoI Head, a lightweight projection head is first used to map it into a low-dimensional embedding space:

(16) $${\tilde{z}}_{i}=\mathrm{Proj}\left(F_{roi}^{i}\right),\;\mathrm{Proj}\left(\cdot \right):FC\to ReLU\to FC.$$

where ${\tilde{z}}_{i}$ denotes the projected RoI embedding feature. Then, $L_{2}$ normalization is applied to reduce the influence of feature magnitude on similarity calculation:

(17) $$z_{i}=\frac{{\tilde{z}}_{i}}{{∥{\tilde{z}}_{i}∥}_{2}}.$$

After normalization, the similarity between any two samples $z_{i}$ and $z_{j}$ is computed by cosine similarity:

(18) $$\mathrm{sim}\left(z_{i},z_{j}\right)=z_{i}^{T}z_{j}.$$

For an object anchor $z_{i}$, its positive sample set consists of object RoIs that belong to the same class and have high overlap with the corresponding ground-truth boxes:

(19) $$P\left(i\right)=\left\{z_{p}|p\neq i,\;y_{p}=y_{i},\;IoU_{p}>\delta_{pos}\right\}.$$

where $y_{i}$ and $y_{p}$ denote the category labels of the anchor and the candidate positive sample, respectively. $IoU_{p}$ denotes the rotated IoU between the candidate RoI and its matched ground-truth box, and $\delta_{pos}$ is the positive overlap threshold.

The negative sample set is composed of hard background samples selected by HBM:

(20) $$N_{hard}\left(i\right)=\left\{z_{n}|r_{n}\in B_{hard}\right\}.$$

Compared with randomly selected background samples, hard background samples usually have higher foreground confidence but lower overlap with real objects, making them closer to actual false-positive sources. Therefore, using them as negative samples in contrastive learning helps the model learn a more fine-grained decision boundary between real objects and confusing backgrounds.

Based on the above positive and negative sample sets, the OBCS Loss for the object anchor $z_{i}$ is defined as

(21) $$L_{obcs}^{i}=-\log\frac{\sum_{z_{p}\in P\left(i\right)}\exp\left(\mathrm{sim}\left(z_{i},z_{p}\right)/\tau \right)}{\sum_{z_{p}\in P\left(i\right)}\exp\left(\mathrm{sim}\left(z_{i},z_{p}\right)/\tau \right)+\sum_{z_{n}\in N_{hard}\left(i\right)}\exp\left(\mathrm{sim}\left(z_{i},z_{n}\right)/\tau \right)}.$$

where τ is the temperature coefficient used to adjust the smoothness of the similarity distribution. Minimizing this loss increases the similarity between the anchor and same-class object samples, while decreasing the similarity between the anchor and hard background samples. The final OBCS Loss is averaged over all valid object anchors:

(22) $$L_{obcs}=\frac{1}{N_{pos}}\sum_{i=1}^{N_{pos}}L_{obcs}^{i}.$$

where $N_{pos}$ denotes the number of valid object anchors participating in contrastive optimization. If the positive sample set of an anchor is empty, this anchor is excluded from the OBCS Loss calculation in the current batch. If no valid object anchor exists in a mini-batch, i.e., $N_{pos}=0$, the OBCS Loss is set to zero for that mini-batch. The network is then optimized using the detection loss and the BDFM auxiliary loss only.

By combining the basic detection loss, the background-decoupled auxiliary loss, and the object–background contrastive suppression loss, the overall training objective of BGD-Net is formulated as

(23) $$L_{total}=L_{det}+\lambda_{bd}L_{bd}+\lambda_{obcs}L_{obcs}.$$

where $L_{bd}$ is the background-decoupled auxiliary loss of BDFM, and $\lambda_{bd}$ and $\lambda_{obcs}$ denote the weights of $L_{bd}$ and OBCS Loss, respectively. $L_{det}$ is the basic oriented detection loss, which consists of the RPN loss and the RoI Head loss:

(24) $$\begin{array}{cc} L_{det} & =L_{rpn}+L_{roi},\;L_{rpn}=L_{rpn}^{cls}+L_{rpn}^{reg},\;L_{roi}=L_{roi}^{cls}+L_{roi}^{reg}. \end{array}$$

After introducing HBM, the RoI classification branch adopts the hard-background reweighted classification loss, namely $L_{roi}^{cls}=L_{cls}^{hbm}$. Therefore, the basic oriented detection loss can be further written as

(25) $$L_{det}=L_{rpn}^{cls}+L_{rpn}^{reg}+L_{cls}^{hbm}+L_{roi}^{reg}.$$

Through joint optimization, BDFM weakens background responses at the feature level, HBM mines high-risk background samples at the sample level, and OBCS Loss enlarges the feature distance between real objects and hard backgrounds in the embedding space. These components collaboratively improve the discriminative ability and detection robustness of the model in complex remote sensing scenes. The projection head and OBCS Loss are used only during training and are removed during inference; therefore, they do not introduce additional computational overhead during testing.

## 4. Experiments and Analysis

### 4.1. Experimental Settings

#### 4.1.1. Datasets

To comprehensively evaluate the effectiveness of the proposed BGD-Net for oriented object detection in complex remote sensing scenes, experiments are conducted on three public datasets: DOTA-v1.0, HRSC2016, and DIOR-R. These datasets are used to evaluate the model from the perspectives of multi-class aerial object detection, harbor ship detection, and cross-dataset generalization under complex backgrounds, respectively.

DOTA-v1.0 is a large-scale aerial remote sensing dataset for oriented object detection. It contains 2806 high-resolution remote sensing images and 15 object categories, including plane (PL), baseball diamond (BD), bridge (BR), ground track field (GTF), small vehicle (SV), large vehicle (LV), ship (SH), tennis court (TC), basketball court (BC), storage tank (ST), soccer-ball field (SBF), roundabout (RA), harbor (HA), swimming pool (SP), and helicopter (HC). Objects in this dataset show arbitrary orientations, large-scale variations, dense distributions, and complex backgrounds, making it suitable for evaluating the comprehensive detection ability of the model in complex remote sensing scenes. Following the official data split, the original images are cropped into 1024×1024 patches with an overlap of 200 pixels to reduce boundary truncation. DOTA-v1.0 is used as the main benchmark for quantitative comparison and ablation studies.

HRSC2016 is a high-resolution remote sensing dataset for ship detection. It mainly contains arbitrarily oriented ships in harbors, offshore areas, and nearshore regions. Although HRSC2016 is a single-class ship detection dataset, ships usually present elongated shapes, large aspect ratios, and diverse orientations. Meanwhile, dock edges, shorelines, ship shadows, and harbor facilities are easily confused with real ships. Therefore, this dataset is suitable for verifying the ability of the proposed method to suppress high-confidence false positives in harbor scenes. The original aspect ratio is preserved during resizing, where the short side is set to 800 pixels and the long side is no larger than 1333 pixels, without extra cropping, to preserve the geometric integrity of ship targets as much as possible.

DIOR-R is the rotated bounding box extension of the DIOR dataset. It contains 20 remote sensing object categories and covers various complex scenes, such as airports, harbors, urban roads, industrial areas, playgrounds, and dense building areas. Compared with DOTA-v1.0, DIOR-R has greater diversity in imaging conditions, spatial resolutions, scene types, and object appearances. It is used to further evaluate the adaptability and generalization ability of the model under different data distributions and complex backgrounds. In this dataset, the network architecture is kept unchanged, and the model is retrained using the same training strategy to verify whether BDFM, HBM, and OBCS Loss can still improve complex background discrimination under different data distributions.

#### 4.1.2. Evaluation Metrics

The mean Average Precision (mAP), which is widely used in oriented object detection, is adopted as the main evaluation metric. For each category, a detection is considered correct according to the rotated IoU between the predicted rotated box and the ground-truth rotated box, and the AP of this category is computed from the precision–recall curve. The mAP over all categories is defined as

(26) $$mAP=\frac{1}{C}\sum_{c=1}^{C}AP_{c},$$

where *C* denotes the number of categories. Different datasets adopt different evaluation protocols. DOTA-v1.0 follows the official evaluation protocol and reports mAP at an IoU threshold of 0.5, denoted as mAP@0.5. For HRSC2016, the VOC2007 11-point interpolation protocol is adopted to compute AP at an IoU threshold of 0.5. Since HRSC2016 contains only one object category, AP@0.5 is numerically equivalent to mAP@0.5; therefore, we report it as mAP@0.5 for consistency throughout the paper. DIOR-R is evaluated using mAP@0.5 under its standard evaluation protocol.

To further analyze the ability of the model to suppress false positives in complex backgrounds, the number of high-confidence hard false positives is counted and denoted as Hard-FP. Specifically, a prediction is counted as a Hard-FP if its confidence score is higher than 0.5 and its maximum rotated IoU with any ground-truth box is below a specified evaluation IoU threshold. Unless otherwise stated, an IoU threshold of 0.30 is used for the main Hard-FP results. To examine whether the evaluation is sensitive to this threshold, additional Hard-FP results under multiple evaluation IoU thresholds are reported in the Section 4.8. In addition, Params and FLOPs are reported in the DOTA-v1.0 comparison to analyze model complexity.

#### 4.1.3. Implementation Details

The proposed method adopts an Oriented R-CNN-style two-stage oriented object detection framework as the baseline detector, with ResNet-50-FPN as the backbone. The main implementation details are shown in Table 1. The input image is first processed by the backbone and FPN to extract multi-scale features. Then, BDFM is inserted between FPN and oriented RPN to enhance object responses and suppress background interference on $\left\{P_{2},P_{3},P_{4},P_{5}\right\}$. After oriented RPN generates rotated proposals, Rotated RoIAlign is used to extract instance-level RoI features, and the RoI Head performs classification and rotated box regression.

During training, HBM calculates the hard background score $H_{i}=p_{i}^{fg}\left(1-IoU_{i}\right)$ according to the foreground confidence $p_{i}^{fg}$ and the maximum rotated IoU of each candidate region, and selects the Top-*K* high-risk background candidates as the hard background set $B_{hard}$. OBCS Loss constructs the positive object set P(i) and the hard background negative set $N_{hard}\left(i\right)$ using the embedding features from the RoI Head, and pulls same-class object features closer while pushing hard background features away.

For the reproduced Oriented R-CNN, ablation experiments, and BGD-Net, the same training and testing settings are adopted. Results of public methods are mainly cited from their original papers. SGD is used as the optimizer, with a momentum of 0.9 and a weight decay of 0.0001. DOTA-v1.0 and DIOR-R are trained for 12 epochs with an initial learning rate of 0.01, which is decayed at the 8th and 11th epochs. HRSC2016 is trained for 36 epochs with an initial learning rate of 0.005.

In BDFM, the background suppression coefficient $\lambda_{bg}$ is set to 0.5, and the learnable residual coefficient γ is initialized to 0 and jointly optimized with the network parameters. On DOTA-v1.0, γ reaches 0.249, 0.364, and 0.386 at epochs 4, 8, and 12, respectively, with progressively smaller changes in the later training stage, indicating a stable convergence tendency.

In HBM, $\delta_{bg}=0.3$ and $\delta_{pos}=0.5$. Top-*K* is set to 128 on DOTA-v1.0 and DIOR-R, and 64 on HRSC2016. For OBCS Loss, the temperature coefficient is set to τ=0.07, the loss weight $\lambda_{obcs}$ is set to 0.1, and the background-decoupled auxiliary loss weight $\lambda_{bd}$ is set to 0.5. During inference, HBM and OBCS Loss are not used, and only BDFM is retained; therefore, the additional inference overhead is limited.

### 4.2. Comparison on DOTA-v1.0

To evaluate the detection performance and model complexity of BGD-Net on the DOTA-v1.0 test set, several representative oriented object detection methods are selected for comparison. To improve table readability, the 15 category-wise AP values are not listed in the main table. Instead, detector type, backbone/configuration, multi-scale setting, parameters, FLOPs, and mAP@0.5 are reported. The table mainly adopts multi-scale training/testing results reported in public papers. The results of BGD-Net are obtained under the same multi-scale setting. Since some methods do not report parameters or computational cost in their original papers, “–” is used for unavailable values.

As shown in Table 2, BGD-Net achieves 82.94% mAP@0.5 with the R-50-FPN backbone, obtaining the best performance among the listed methods. Compared with the baseline detector Oriented R-CNN, BGD-Net improves mAP@0.5 from 80.87% to 82.94%, with a gain of 2.07 percentage points. Since both methods use the same backbone, pre-training strategy, and multi-scale testing setting, this improvement directly demonstrates the effectiveness of the proposed background-decoupled learning mechanism.

In terms of model complexity, BGD-Net has 42.1M parameters and 204G FLOPs. Compared with Oriented R-CNN, it only introduces 1.0M additional parameters and 5G additional FLOPs. In other words, BGD-Net brings only about 2.43% parameter increase and 2.51% computational increase, while achieving a 2.07 percentage-point accuracy improvement. This result indicates that the proposed method can effectively improve oriented object detection performance in complex remote sensing scenes with limited additional computational cost.

Moreover, BGD-Net outperforms KFIoU, AOPG, RTMDet-R, and LSKNet-S by 2.01, 2.28, 2.40, and 1.30 percentage points, respectively, showing strong competitiveness in complex background scenes. Considering both accuracy and complexity, BGD-Net does not rely on a stronger backbone or a larger model scale. Instead, it alleviates object–background confusion through BDFM, HBM, and OBCS Loss from the feature, sample, and optimization levels, thereby achieving more robust detection performance.

To further evaluate practical inference efficiency, we measure the inference speed of Oriented R-CNN and BGD-Net under the same hardware and software environment. All experiments are conducted on a single NVIDIA Tesla T4 GPU with a batch size of 1 and an input size of 1024×1024. After 100 warm-up iterations, 500 inference runs are performed with CUDA synchronization, and the average inference speed is reported in frames per second (FPS).

As shown in Table 3, BGD-Net achieves an inference speed of 19.65 FPS, compared with 20.58 FPS for Oriented R-CNN. Together with the small increases in parameters and FLOPs, this result indicates that BGD-Net maintains comparable practical inference efficiency while improving detection accuracy.

### 4.3. Comparison on HRSC2016

To verify the robustness of the proposed method under harbor complex backgrounds, experiments are further conducted on the HRSC2016 ship detection dataset. Table 4 reports the ship detection results of different oriented object detection methods on HRSC2016. BGD-Net achieves 91.42% mAP@0.5 on HRSC2016, outperforming the Oriented R-CNN baseline of 90.50% by 0.92 percentage points. This result demonstrates that the proposed method remains effective for oriented ship detection under complex harbor backgrounds. Since HRSC2016 is close to performance saturation and the accuracy gaps among advanced methods are relatively small, this improvement indicates that the proposed method still provides stable gains under harbor complex backgrounds.

Ships in HRSC2016 usually have elongated shapes, diverse orientations, dense berthing patterns, and strong background interference. Dock edges, shipyard structures, shorelines, and shadow regions can be easily confused with real ships. BGD-Net suppresses complex background responses through BDFM, focuses on high-confidence hard background samples through HBM, and enlarges the feature distance between objects and hard backgrounds using OBCS Loss. Therefore, it further improves ship detection performance.

### 4.4. Generalization on DIOR-R

To evaluate the cross-dataset generalization ability of BGD-Net under complex background scenes, experiments are conducted on DIOR-R. DIOR-R contains various complex remote sensing scenes, including airports, harbors, urban roads, industrial areas, and dense buildings. The object appearances and background structures are more diverse, making it suitable for evaluating model generalization. Table 5 shows the comparison results of different oriented object detection methods on DIOR-R. Since the original Oriented R-CNN paper does not report results on DIOR-R, Oriented R-CNN is reproduced under the same experimental setting and used as the baseline detector.

As shown in Table 5, BGD-Net achieves 68.35% mAP@0.5, outperforming all compared methods. Compared with the reproduced Oriented R-CNN, BGD-Net improves mAP@0.5 from 64.36% to 68.35%, with a gain of 3.99 percentage points. In addition, BGD-Net also outperforms Oriented RepPoints, DCFL, and RQFormer, indicating that the proposed method has good generalization ability under different data distributions.

This result shows that BGD-Net is not only effective on DOTA-v1.0. Through the collaborative effect of BDFM, HBM, and OBCS Loss, the model can better distinguish real objects from complex backgrounds, thereby improving cross-dataset-oriented object detection performance in remote sensing images.

### 4.5. Ablation Study

To verify the effectiveness of BDFM, HBM, and OBCS Loss, ablation experiments are conducted on DOTA-v1.0 using Oriented R-CNN as the baseline detector. The results are shown in Table 6. In addition to mAP@0.5, Hard-FP is further counted to measure the number of high-confidence false positives in complex background regions. A lower Hard-FP indicates stronger suppression ability against false positives caused by complex backgrounds.

As shown in Table 6, all three modules bring stable improvements. When BDFM is added alone, mAP@0.5 increases from 80.87% to 81.68%, and Hard-FP decreases from 1128 to 926, indicating that BDFM can enhance object responses and weaken complex background interference. When HBM is added alone, mAP@0.5 increases to 81.32%, and Hard-FP decreases to 884, demonstrating that hard background mining can effectively reduce high-confidence background false positives. When OBCS Loss is added alone, mAP@0.5 reaches 81.41%, showing that object–background contrastive constraints can enhance the discriminability of RoI features.

When the three modules are jointly used, BGD-Net obtains the best result, achieving 82.94% mAP@0.5, which is 2.07 percentage points higher than Oriented R-CNN. Meanwhile, Hard-FP decreases to 694, corresponding to a reduction of about 38.48%. This demonstrates that BDFM, HBM, and OBCS Loss collaboratively alleviate object–background confusion from the feature, sample, and optimization levels.

Table 7 further verifies the advantage of BGD-Net on complex-background categories. Compared with Oriented R-CNN, the average AP of complex-background categories increases from 78.03% to 80.40%, with a gain of 2.37 percentage points. In particular, BGD-Net achieves clear improvements on categories that are easily affected by background structures, such as bridge, small vehicle, ship, soccer-ball field, roundabout, and harbor. This result indicates that the proposed method can effectively reduce false positives caused by complex backgrounds, such as dock edges, road textures, building structures, and circular facilities.

Overall, the ablation study demonstrates the effectiveness of BDFM, HBM, and OBCS Loss. The best performance is achieved when all three components are combined, verifying the rationality of the object–background decoupled learning mechanism in BGD-Net.

### 4.6. Counterfactual Substitution Experiments

To further verify whether the specific designs of BDFM, HBM, and OBCS Loss are more effective than conventional alternatives, we conduct counterfactual substitution experiments on DOTA-v1.0. In each experiment, only one proposed component is replaced while the remaining network architecture, training strategy, data augmentation, and testing settings are kept unchanged. Specifically, BDFM is replaced with CBAM [37], HBM is replaced with OHEM [42], and OBCS Loss is replaced with standard supervised contrastive loss (SupCon) [46].

As shown in Table 8, all three standard substitution variants improve over the Oriented R-CNN baseline, but remain inferior to the complete BGD-Net. Replacing BDFM with CBAM decreases mAP@0.5 from 82.94% to 82.28%, indicating that generic attention-based feature enhancement cannot fully replace the explicit object–background modeling of BDFM. Similarly, replacing HBM with OHEM results in 82.16% mAP@0.5, suggesting that selecting hard samples solely according to classification loss is less effective than jointly considering foreground confidence and rotated IoU.

When OBCS Loss is replaced with standard supervised contrastive learning, the model achieves 82.33% mAP@0.5, which is lower than the 82.94% achieved by the complete BGD-Net. These results indicate that the performance improvement is not merely caused by introducing attention, hard-example mining, or contrastive learning in a generic form. Instead, the specific formulations of BDFM, HBM, and OBCS are better suited to suppressing object–background confusion in complex remote sensing scenes.

### 4.7. Statistical Robustness Analysis

To evaluate the robustness of the reported improvements against random initialization, we repeated the baseline and the main BGD-Net variants using three independent random seeds under identical training and evaluation settings. For each method, we report the mean and standard deviation of mAP@0.5 and Hard-FP. The same set of random seeds was used for all compared methods to reduce the influence of seed-dependent variation.

As shown in Table 9, BGD-Net achieves an average mAP@0.5 of 82.93 ± 0.07% across three independent runs, compared with 80.87 ± 0.08% for the Oriented R-CNN baseline. Meanwhile, the average Hard-FP is reduced from 1126.7 ± 14.6 to 695.0 ± 9.5. The relatively small standard deviations across the three runs indicate that the performance improvements are reproducible under different random initializations.

### 4.8. Hyperparameter Analysis

**Figure 4:**
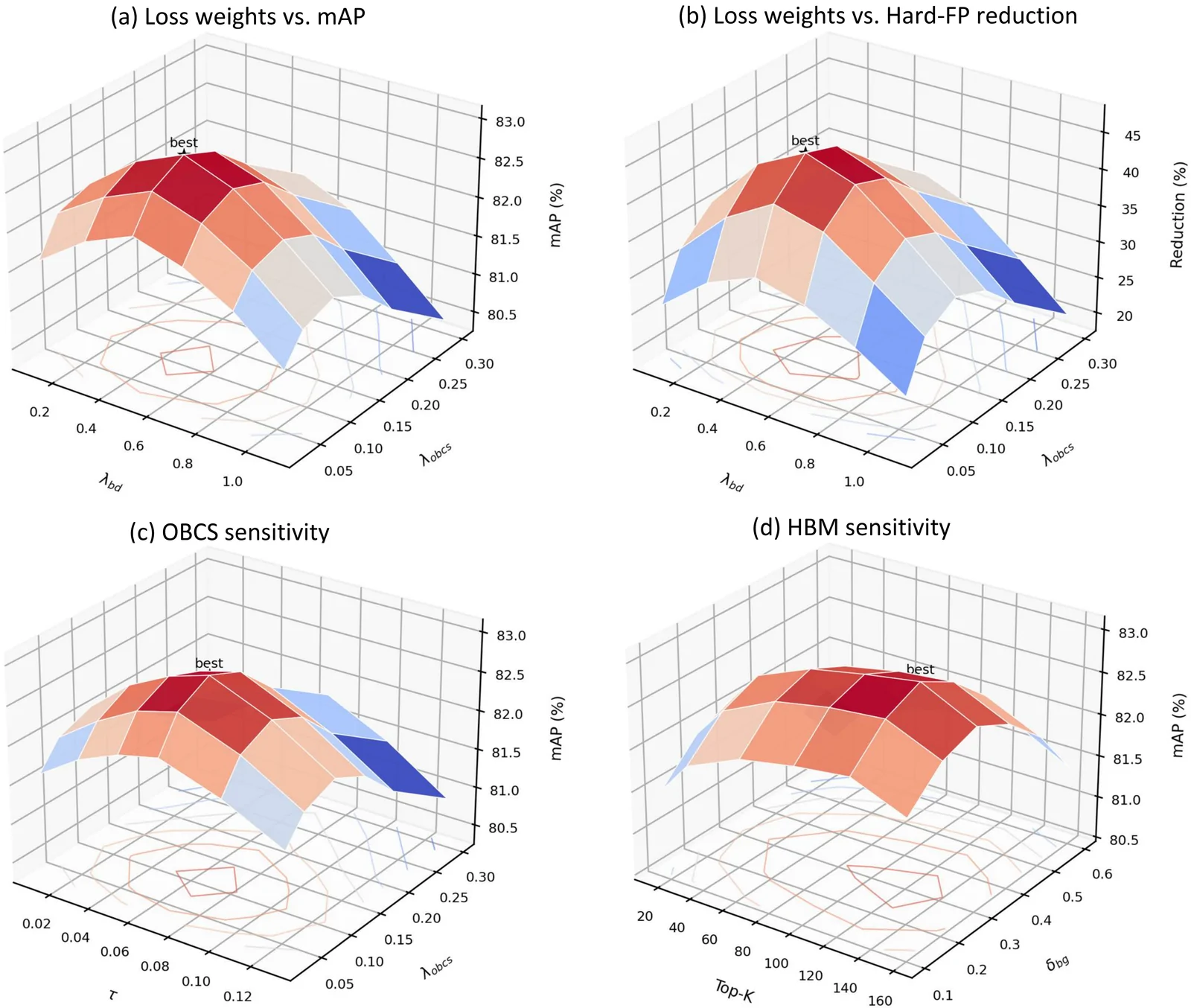
Parameter sensitivity analysis of BGD-Net on DOTA-v1.0.

To analyze the influence of key hyperparameters on BGD-Net, parameter sensitivity experiments are conducted on DOTA-v1.0. A controlled-variable strategy is adopted, where only the corresponding parameters in each experiment are changed, while the network architecture, training strategy, and post-processing settings remain unchanged. The analyzed parameters include the background-decoupled auxiliary loss weight $\lambda_{bd}$, the object–background contrastive suppression loss weight $\lambda_{obcs}$, the temperature coefficient τ, the hard background sampling number Top-*K*, and the background threshold $\delta_{bg}$. The results are shown in Figure 4.

Figure 4a,b show the joint influence of $\lambda_{bd}$ and $\lambda_{obcs}$ on mAP@0.5 and Hard-FP reduction, respectively. Hard-FP reduction denotes the reduction ratio of hard false positives compared with the Oriented R-CNN baseline. When $\lambda_{bd}=0.5$ and $\lambda_{obcs}=0.10$, the model achieves the highest mAP@0.5 of 82.94%, while Hard-FP decreases from 1128 to 694, corresponding to a reduction of 38.48%. When the loss weights are too small, the auxiliary supervision and contrastive constraints are insufficient. When the weights are too large, they may interfere with the optimization of the basic classification and regression tasks.

Figure 4c analyzes the influence of the internal OBCS Loss parameters τ and $\lambda_{obcs}$. The model achieves the best performance when τ=0.07 and $\lambda_{obcs}=0.10$. A smaller τ makes the similarity distribution too sharp, causing gradients to concentrate on only a few hard samples. In contrast, a larger τ over-smooths the similarity distribution and weakens the feature discrimination between real objects and hard backgrounds.

Figure 4d shows the influence of Top-K and $\delta_{bg}$ on HBM. The best result is obtained when Top-K = 128 and $\delta_{bg}=0.30$. A too-small Top-K provides insufficient hard-background samples, whereas a too-large Top-K may introduce redundant or noisy samples. Similarly, a smaller $\delta_{bg}$ may exclude informative hard-background candidates, while a larger threshold may incorrectly include ambiguous or inaccurately localized object proposals in the background set.

Overall, the response surfaces generally increase toward the optimal region and then decrease or become saturated. This indicates that excessively small parameter values provide insufficient constraints, whereas overly large values may introduce excessive suppression or noise interference. Meanwhile, the relatively smooth variations around the optimum indicate that the selected parameters provide a reasonable operating region. Based on these experiments, the final settings are $\lambda_{bd}=0.5$, $\lambda_{obcs}=0.10$, τ=0.07, Top-K = 128, and $\delta_{bg}=0.30$.

It should be noted that $\delta_{bg}=0.30$ is used for background candidate selection during HBM training. To further examine whether the observed Hard-FP reduction is specific to the IoU threshold of 0.30, we keep the trained models fixed and recalculate Hard-FP using different evaluation IoU thresholds of 0.10, 0.20, 0.30, 0.40, and 0.50. No model retraining or parameter tuning is performed during this evaluation. The corresponding results are reported in Table 10.

As shown in Table 10, BGD-Net consistently produces fewer Hard-FPs than the Oriented R-CNN baseline across all tested evaluation IoU thresholds. The Hard-FP reduction remains approximately 37–38% when the evaluation IoU threshold varies from 0.10 to 0.50. Importantly, the improvement is not restricted to the threshold of 0.30 used during HBM training. These results demonstrate that the reduction in high-confidence background false positives is maintained under different evaluation criteria.

### 4.9. Visualization Analysis

**Figure 5:**
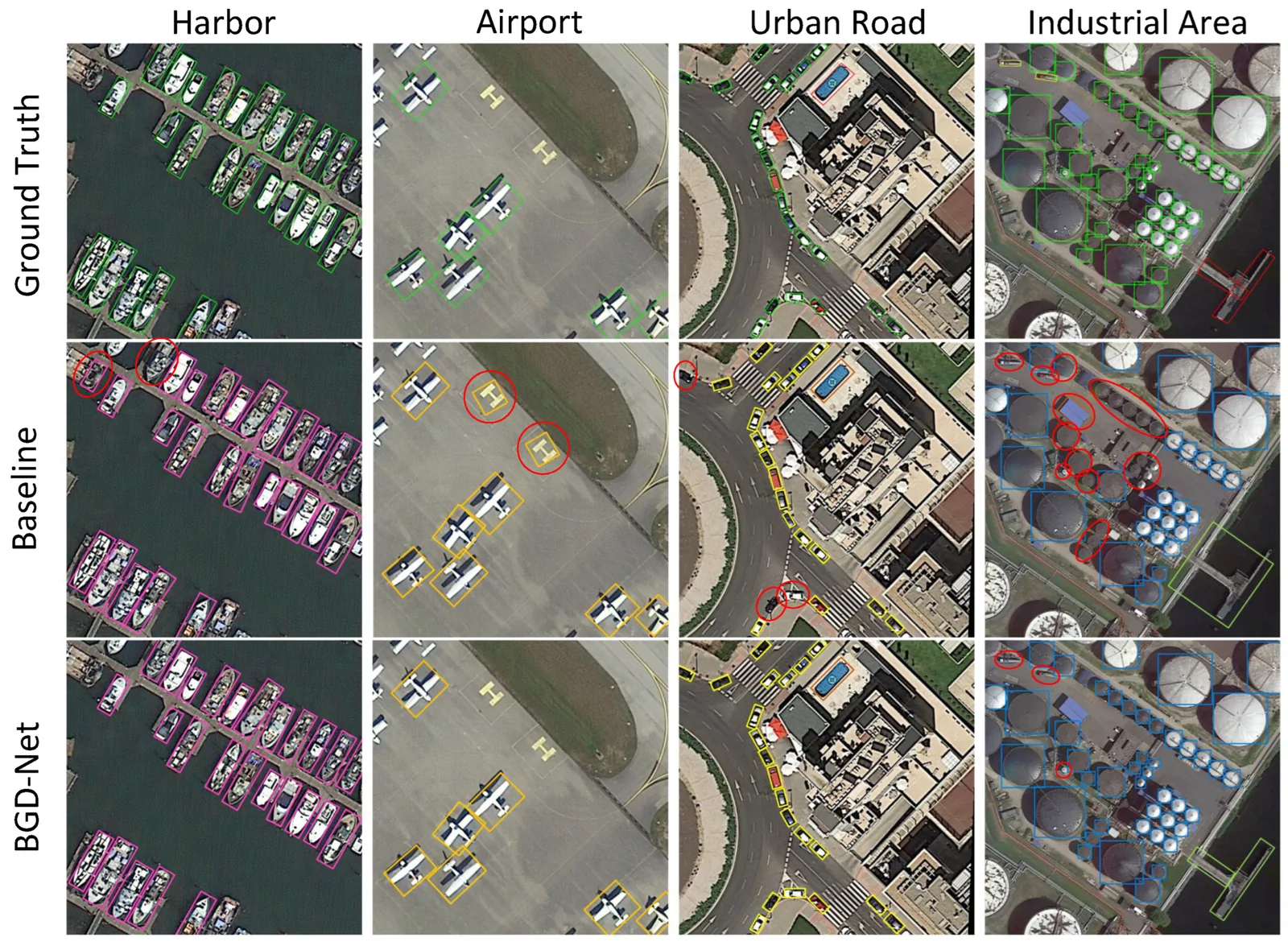
Detection result comparison between Oriented R-CNN and BGD-Net under complex background scenes. From left to right: harbor, airport, urban road, and industrial area scenes. From top to bottom: ground truth, Oriented R-CNN baseline results, and BGD-Net detection results. Red circles indicate missed detections or false positives.

To intuitively verify the detection performance of BGD-Net in complex background scenes, harbor, airport, urban road, and industrial area scenes are selected for visualization comparison, as shown in Figure 5. The first row shows the ground-truth annotations, and the second and third rows show the detection results of the Oriented R-CNN baseline and BGD-Net, respectively. Red circles indicate missed detections or false positives.

In the harbor scene, ships are densely berthed and close to dock edges. The baseline method is easily affected by pier edges and neighboring ships, resulting in missed detections or incomplete localization near image boundaries and dense regions. BGD-Net can more completely detect ships with different orientations and scales, and reduce false predictions caused by complex dock backgrounds. In the airport scene, the baseline method misclassifies some “H”-shaped ground markings as aircraft, while BGD-Net effectively suppresses such regular geometric background interference and retains real aircraft targets.

In the urban road scene, small vehicles are densely distributed along curved roads and intersections. The baseline method is affected by road markings, vehicle shadows, and building edges, producing some missed detections and false positives. BGD-Net detects vehicles along roadsides more continuously and stably. In the industrial area, storage tanks, building roofs, and other circular industrial structures have similar geometric appearances. The baseline method has more missed detections for small-scale and low-contrast objects. BGD-Net can more accurately localize storage tanks of different scales and reduce false positives caused by complex industrial backgrounds, although a few errors still exist in extremely dense or boundary-ambiguous regions.

Overall, BGD-Net shows better detection stability in different complex scenes. It especially alleviates object–background confusion caused by dock structures, airport markings, road textures, and industrial facilities. The visualization results are consistent with the quantitative and ablation results, indicating that the proposed background-decoupled learning strategy can effectively reduce missed detections and high-confidence false positives caused by complex backgrounds, and improve the adaptability of the model to dense objects, small-scale objects, and regular geometric background interference.

## 5. Discussion

The experimental results demonstrate that BGD-Net effectively alleviates object–background confusion in complex remote sensing scenes through coordinated optimization at the feature, sample, and embedding levels. Compared with the baseline, the proposed method consistently reduces high-confidence false positives while improving oriented object detection performance across multiple datasets. These results suggest that explicitly modeling background interference is beneficial for complex-scene oriented object detection.

The ablation results further show that BDFM, HBM, and OBCS Loss provide complementary contributions. BDFM reduces background interference before proposal generation, HBM emphasizes high-risk background regions during training, and OBCS Loss improves the separation between object and hard-background representations in the embedding space. Their combined use therefore improves object–background discrimination without relying solely on increased model capacity.

Nevertheless, the proposed method still has some limitations. First, the background threshold and Top-*K* value in HBM, as well as the temperature coefficient and loss weights in OBCS Loss, still need to be set according to different datasets. Second, OBCS Loss depends on candidate regions generated by the preceding stage. When very small objects, heavily occluded objects, or low-contrast objects are not effectively recalled, the subsequent contrastive constraint cannot fully compensate for the insufficient proposals. In addition, the current experiments are mainly conducted on optical remote sensing images and a two-stage detection framework. Its applicability to SAR images, multispectral data, and other detection frameworks still requires further verification.

HBM and OBCS Loss are used only during training, while BDFM is mainly retained during inference. Therefore, the additional inference overhead is limited. Future work will explore adaptive hard background selection, enhanced recall for very small objects, and cross-domain generalization. More fine-grained false-positive analysis will also be considered to further improve the adaptability of the model under different imaging conditions and complex scenes.

## Figures and Tables

**Table 1:** Main implementation details.

Setting	DOTA-v1.0	HRSC2016	DIOR-R
Detector	Oriented R-CNN	Oriented R-CNN	Oriented R-CNN
Backbone	ResNet-50-FPN	ResNet-50-FPN	ResNet-50-FPN
Input size	1024×1024	short side 800, max long side 1333	800×800
Optimizer	SGD	SGD	SGD
Initial learning rate	0.01	0.005	0.01
Epochs	12	36	12
Momentum	0.9	0.9	0.9
Weight decay	0.0001	0.0001	0.0001
$\delta_{bg}/\delta_{pos}$	0.3/0.5	0.3/0.5	0.3/0.5
Top-*K* in HBM	128	64	128
τ in OBCS	0.07	0.07	0.07
$\lambda_{bd}/\lambda_{obcs}$	0.5/0.1	0.5/0.1	0.5/0.1
$\lambda_{bg}$	0.5	0.5	0.5
α in HBM	1.0	1.0	1.0
γ initialization	0	0	0
Projection embedding dim.	128	128	128
Batch size per GPU	2	2	2

**Table 2:** Comparison of detection performance and model complexity on the DOTA-v1.0 test set.

Method	Type	Backbone/Config	Pretrain	MS	Params (M)	FLOPs (G)	mAP@0.5 (%)
R3Det [3]	One-stage	R-50-FPN	IN	Yes	41.9	336	76.47
CFA [50]	One-stage	R-50-FPN	IN	Yes	–	–	76.67
S2A-Net [4]	One-stage	R-50-FPN	IN	Yes	38.6	198	79.42
R3Det-GWD [3,7]	One-stage	R-50-FPN	IN	Yes	41.9	336	80.23
RTMDet-R [51]	One-stage	RTMDet-R	IN	Yes	52.3	205	80.54
R3Det-KLD [3,8]	One-stage	R-50-FPN	IN	Yes	41.9	336	80.63
RTMDet-R [51]	One-stage	RTMDet-R	CO	Yes	52.3	205	81.33
RoI Transformer [1]	Two-stage	R-50-FPN	IN	Yes	55.1	200	74.61
Gliding Vertex [25]	Two-stage	R-50-FPN	IN	Yes	41.1	198	75.02
CSL [5]	One-stage	R-50-FPN	IN	Yes	37.4	236	76.17
ReDet [24]	Two-stage	ReR50	IN	Yes	31.6	–	80.10
DODet [26]	Two-stage	R-50-FPN	IN	Yes	–	–	80.62
AOPG [27]	Two-stage	R-50-FPN	IN	Yes	–	–	80.66
Oriented R-CNN [2]	Two-stage	R-50-FPN	IN	Yes	41.1	199	80.87
KFIoU [28]	Two-stage	R-50-FPN	IN	Yes	58.8	206	80.93
LSKNet-S [29]	Two-stage	LSKNet-S	IN	Yes	31.0	161	81.85
**BGD-Net (Ours)**	**Two-stage**	**R-50-FPN**	**IN**	**Yes**	**42.1**	**204**	**82.94**

*Note:* Type denotes the detector type. Pretrain denotes the pre-training dataset, where IN and CO indicate ImageNet and COCO pre-training, respectively. MS denotes multi-scale training/testing. Params and FLOPs represent the number of parameters and computational cost, respectively. FLOPs are calculated with an input size of 1024×1024. mAP@0.5 denotes the mean average precision at an IoU threshold of 0.5. “–” indicates that the result is not reported in the original paper.

**Table 3:** Empirical inference efficiency of Oriented R-CNN and BGD-Net on DOTA-v1.0.

Method	Input	Params (M)	FLOPs (G)	FPS
Oriented R-CNN	1024×1024	41.1	199	20.58
BGD-Net (Ours)	1024×1024	42.1	204	19.65

**Table 4:** Ship detection results on the HRSC2016 dataset.

Method	Backbone	mAP@0.5 (%)
R2CNN [52]	ResNet-50	73.07
RRPN [23]	ResNet-101	79.08
RoI Transformer [1]	ResNet-101	86.20
Gliding Vertex [25]	ResNet-101	88.20
S2A-Net [4]	ResNet-50	90.17
R3Det [3]	ResNet-50	89.26
ReDet [24]	ReR50	90.46
Oriented R-CNN [2]	ResNet-50-FPN	90.50
KFIoU [28]	ResNet-50	90.65
LSKNet-S [29]	LSKNet-S	90.71
**BGD-Net (Ours)**	**ResNet-50-FPN**	**91.42**

*Note: All results are evaluated at an IoU threshold of 0.5. For HRSC2016, AP is calculated using the VOC2007 11-point evaluation protocol.*

**Table 5:** Generalization results on the DIOR-R dataset.

Method	Type	Backbone	mAP@0.5 (%)
RetinaNet-O [43]	One-stage	R-50-FPN	57.55
Faster R-CNN-O [53]	Two-stage	R-50-FPN	59.54
Gliding Vertex [25]	Two-stage	R-50-FPN	60.06
RoI Transformer [1]	Two-stage	R-50-FPN	63.87
AOPG [27]	Two-stage	R-50-FPN	64.41
Oriented R-CNN (reproduced) [2]	Two-stage	R-50-FPN	64.36
Oriented RepPoints [30]	One-stage	R-50-FPN	66.71
DCFL [31]	One-stage	R-50	66.80
RQFormer [32]	End-to-end	R-50	67.31
**BGD-Net (Ours)**	**Two-stage**	**R-50-FPN**	**68.35**

*Note:* “reproduced” denotes results reproduced by us under the same training and evaluation protocol. mAP@0.5 denotes the mean average precision at an IoU threshold of 0.5.

**Table 6:** Ablation study of BDFM, HBM, and OBCS Loss. ↓ indicate that lower values are better.

Method	BDFM	HBM	OBCS	mAP@0.5 (%)	Hard-FP ↓
Baseline				80.87	1128
Baseline + BDFM	√			81.68	926
Baseline + HBM		√		81.32	884
Baseline + OBCS			√	81.41	941
Baseline + BDFM + HBM	√	√		82.21	782
Baseline + BDFM + OBCS	√		√	82.34	805
Baseline + HBM + OBCS		√	√	82.06	816
**BGD-Net (Full)**	√	√	√	**82.94**	**694**

**Table 7:** Ablation results on complex-background categories. Unit: AP (%).

Method	BR	SV	LV	SH	ST	SBF	RA	HA	HC	Mean
Oriented R-CNN	61.09	79.71	85.35	88.82	87.73	72.21	70.80	82.42	74.11	78.03
+BDFM	62.46	81.05	86.22	89.36	88.26	73.82	72.11	83.58	75.62	79.16
+HBM	62.03	80.73	85.96	89.18	88.05	73.45	71.78	83.21	75.31	78.86
+OBCS Loss	62.20	80.86	86.08	89.27	88.12	73.66	72.04	83.34	75.48	79.01
**BGD-Net**	**64.10**	**82.75**	**87.10**	**90.02**	**88.86**	**75.34**	**73.52**	**84.75**	**77.18**	**80.40**

**Table 8:** Counterfactual substitution experiments on DOTA-v1.0.

Method	Feature Module	Sample Selection	Contrastive Loss	mAP@0.5 (%)
Baseline	–	Standard	–	80.87
BGD-Net w/CBAM	CBAM	HBM	OBCS	82.28
BGD-Net w/OHEM	BDFM	OHEM	OBCS	82.16
BGD-Net w/SupCon	BDFM	HBM	SupCon	82.33
**BGD-Net (Full)**	**BDFM**	**HBM**	**OBCS**	**82.94**

**Table 9:** Statistical robustness over three independent runs on DOTA-v1.0. ↑ and ↓ indicate that higher and lower values are better, respectively.

Method	Runs	mAP@0.5 (%) ↑	Hard-FP ↓
Oriented R-CNN	3	80.87±0.08	1126.7±14.6
+BDFM	3	81.67±0.10	928.0±10.4
+HBM	3	81.31±0.07	886.3±15.0
+OBCS	3	81.40±0.09	939.7±11.2
**BGD-Net (Full)**	**3**	82.93±0.07	695.0±9.5

**Table 10:** Hard-FP results under different evaluation IoU thresholds on DOTA-v1.0.

Evaluation IoU Threshold	Oriented R-CNN	BGD-Net	Reduction (%)
0.10	725.3±11.2	455.0±8.0	37.27
0.20	924.7±12.8	576.3±8.7	37.68
0.30	1126.7±14.6	695.0±9.5	38.32
0.40	1368.0±16.2	842.7±11.0	38.40
0.50	1652.3±18.0	1029.0±13.2	37.72

## Data Availability

We clarify that our research findings are based on the analysis of publicly available datasets: DOTA-v1.0: https://captain-whu.github.io/DOTA/dataset.html (accessed on 19 December 2025). HRSC2016: https://ieee-dataport.org/documents/hrsc2016-0 (accessed on 20 December 2025). DIOR-R: https://gcheng-nwpu.github.io/ (accessed on 29 January 2026). The source code of OPT-Net is publicly available at https://github.com/jj0523/BGD-Net-MMRotate (accessed on 9 August 2026).
